# Out-Patients in Hospitals

**Published:** 1890-09-27

**Authors:** 


### Editor's Letter-Box.

[Our Correspondents are reminded that prolixity is a great bar to publication, and that brevity of style and conciseness of statement greatly facilitate early insertion.]

### OUT-PATIENTS IN HOSPITALS.

Sir,—Having a long and considerable knowledge of the poorer classes as well as of the "Provident Medical Association" alluded to in your article, I should like to notice one or two matters which I believe are not generally understood with regard to both subjects.

1. I would say that the extremely low rates of payment in the provident dispensaries bring them within reach of almost, if not quite, all those who are not classed as the "destitute," and who thus come within the range of the poor law, under which it is not generally known excellent dispensaries are established in every metropolitan union.

2. I would ask if all, or nearly all, those who plead inability to pay anything do not spend far more than 6d. a-month (more probably in a week or a day) in beer or other unnecessary stimulant? Do not the children of the poorest get pence to spend in sweets and ices throughout the week, and why, if thrift were thought of, and not wholly discouraged by our present plans, should not some at least of all this expenditure be bestowed on providing for the certain and inevitable misfortune of sickness? The answer is clear; because by our plans for past generations no inducement is given for any such expenditure, and is directly discouraged by all our treatment of the poor. You rightly say that "many of those who might be provident are not, and never will be made so." I may add, I fear not, under our present system of throwing free medical attendance before all the working classes. It is true that help cannot be denied at the moment of sickness, but is that any reason why we should not all the more earnestly endeavour to educate present and future generations in the perfectly possible virtue of thrift and foresight?

The "indifference" of the poor can never be overcome as long as they are subjected to the demoralising and pauperising influences of free medical attendance, of which even the well to do and those in the public service do not scruple to avail themselves. L.

September 20th.

P.S. It is a fact that the children of the very poor (so called) spend 2s. or 3s. when taken out for a school treat.

